# The Use of Carbohydrate Biopolymers in Plant Protection against Pathogenic Fungi

**DOI:** 10.3390/polym14142854

**Published:** 2022-07-13

**Authors:** Grażyna Korbecka-Glinka, Klaudia Piekarska, Maria Wiśniewska-Wrona

**Affiliations:** 1 Department of Plant Breeding and Biotechnology, Institute of Soil Science and Plant Cultivation-State Research Institute, Czartoryskich 8, 24-100 Puławy, Poland; 2 Biomedical Engineering Center, Łukasiewicz Research Network-Łódź Institute of Technology, Skłodowskiej-Curie 19/27, 90-570 Łódź, Poland; klaudia.piekarska@lit.lukasiewicz.gov.pl (K.P.); maria.wisniewska-wrona@lit.lukasiewicz.gov.pl (M.W.-W.)

**Keywords:** phytopathogenic fungi, polysaccharides, plant protection, antifungal coatings, seed coating, seed treatments, field applications, pre-harvest treatments, post-harvest treatments, edible coatings

## Abstract

Fungal pathogens cause significant yield losses of many important crops worldwide. They are commonly controlled with fungicides which may have negative impact on human health and the environment. A more sustainable plant protection can be based on carbohydrate biopolymers because they are biodegradable and may act as antifungal compounds, effective elicitors or carriers of active ingredients. We reviewed recent applications of three common polysaccharides (chitosan, alginate and cellulose) to crop protection against pathogenic fungi. We distinguished treatments dedicated for seed sowing material, field applications and coating of harvested fruits and vegetables. All reviewed biopolymers were used in the three types of treatments, therefore they proved to be versatile resources for development of plant protection products. Antifungal activity of the obtained polymer formulations and coatings is often enhanced by addition of biocontrol microorganisms, preservatives, plant extracts and essential oils. Carbohydrate polymers can also be used for controlled-release of pesticides. Rapid development of nanotechnology resulted in creating new promising methods of crop protection using nanoparticles, nano-/micro-carriers and electrospun nanofibers. To summarize this review we outline advantages and disadvantages of using carbohydrate biopolymers in plant protection.

## 1. Introduction

Agriculture today faces a challenge of having to produce food for the growing human population, while pests and pathogens constantly reduce the crop. Global estimates of yield losses caused by pests and pathogens in five major food crops (including wheat, rice, maize, potato and soybean) range from 17.2% to 30.0% [1]. Among the pathogens, fungi and oomycetes are considered to be the most destructive [2,3]. Key aspects of biology of these organisms, important from the epidemiological perspective, include broad host ranges, high virulence, high reproductive potential and ability to survive outside the plant host as a saprophyte or durable spores [3]. Fungal and oomycete pathogens pose a growing threat to the global food security because they spread to new areas with trade and transport or due to climate change. Moreover, common agricultural practices do not help to combat epidemics, as genetically uniform crops are grown in large areas in monocultures while their protection relies on single resistance genes in the plants or/and single-target-site fungicides. Selection pressures in such agroecosystems favor prolific variants of fungicide-resistant pathogens which are able to overcome plant resistance [4].

Introduction of first synthetic organic fungicides (thiram, zineb and nabam) in the 1940s initiated a rapid development of the plant protection industry. In the next three decades, many new active compounds representing major classes of fungicides were produced and applied to plant protection, first to horticultural crops and then to cereals [5]. Soon fungicide treatments became a common practice in agriculture and they were associated with a significant increase in yield, ranging from 14% to 100% depending on the crop [6]. The current list of fungal control agents includes over 230 compounds, and the development of new ones is driven by fungicide resistance management [7,8]. However, widespread usage of pesticides was also associated with the contamination of terrestrial and aquatic ecosystems, toxic effects on non-target organisms and negative impact on human health [9,10]. The growing concerns about these problems have led to introducing regulations concerning safe and efficient use of these agrochemicals and a registration of new active ingredients worldwide [11]. A recent “Farm to Fork Strategy” adopted by the European Commission aims at 50% reduction in the use of chemical pesticides by 2030 [12].

Few alternative approaches were proposed to address the challenge of significant reduction in pesticide use. Lázaro et al. [13] suggested, based on their meta-analysis, that 50% reduction in fungicide use can be achieved by employing decision support systems, which will help the farmers to plan fungicide application based on an observed or a predicted risk of fungal disease. Nevertheless, the agrochemical industry responds to the challenges in plant protection differently-by exploring other two alternative approaches: developing advanced types of fungicides with novel modes of action and improving application of conventional fungicides by means of targeted delivery systems based on encapsulation technology [14]. Another approach is to search for ingredients of safer plant protection formulations among metal/metal oxide nanoparticles, plant extracts, essential oils, antagonistic microorganisms or food additives (e.g., [14,15]).

Carbohydrate biopolymers can also be used to develop plant protection products which will form an alternative to conventional fungicides. These biopolymers can be obtained in large amounts from many natural sources. They are also non-toxic and biodegradable, and therefore suitable for use in organic agriculture. Moreover, they can interact with many hydrophobic and hydrophilic compounds in more complex formulations [16]. There are three functions carbohydrate biopolymers may play in plant protection against pathogenic fungi. Firstly, they may directly interact with fungi by inhibiting spore germination and mycelial growth, what was shown in case of chitosan [17,18]. Secondly, they may act as effective elicitors inducing the plant immune system to cope with pathogens [19]. Thirdly, they may be used as carrier in controlled-release formulations of agrochemicals or other active ingredients [16].

**Figure 1:**
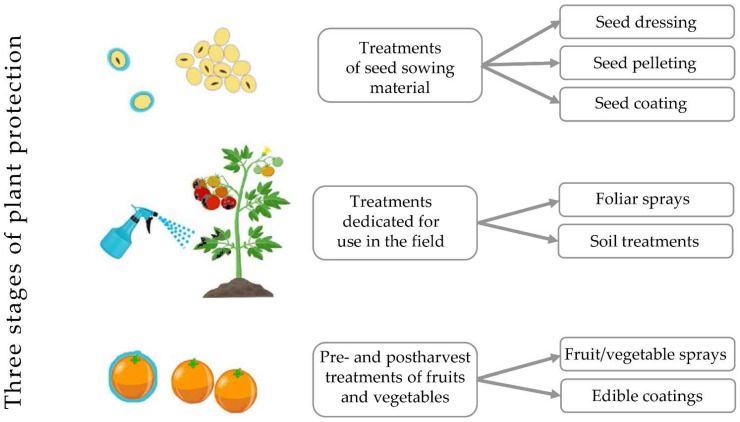
Stages and forms of antifungal plant protection applications which can be based on carbohydrate biopolymers.

Plants are threatened by fungi at different stages of their growth, hence different types of treatments were developed to ensure efficient plant protection (Figure 1). Firstly, seeds may be colonized by pathogenic fungi or they cope with them during germination in the soil. Therefore, various seed treatments were developed to enhance the quality of seed sowing material and to improve plant emergence in the field [20,21,22]. Secondly, in the field, plants are exposed to a variety of air- and soil-borne pathogens, hence antifungal formulations are applied in a form of foliar sprays or soil treatments in order to provide adequate protection [23,24]. Finally, ripe fruits and vegetables may be colonized by fungi which decrease their storability and induce decay; therefore, additional plant protection is required before or after harvest and it is frequently applied in a form of edible coatings [25,26].

Here we aim at reviewing recent studies on using carbohydrate polymers in antifungal formulations dedicated for the above-mentioned stages of crop production, for: seed treatments, use in the field and treatments of harvested crops. We will focus on three commonly available biopolymers: chitosan, alginate and cellulose.

## 2. Chitosan

Chitin is the second most abundant renewable biopolymer in the world [27]. It occurs in marine shellfish, insects, mushrooms and yeast. The highest percentage content of chitin has been observed in shells and tails of crabs, shrimps and lobsters [28]. The best-known derivative of chitin is chitosan, which is a polycationic polymer isolated after the deacetylation of chitin. Chitosan is a linear polymer β-(1→4)-linked D-glucosamine and N-acetyl-D-glucosamine. Compared to chitin, chitosan is more functional due to its amino based functional groups stretching along the chain [29]. In addition, the protonation intensity of amino groups also plays a vital role in its functionality. Following the process of deacetylation, chitosan can be obtained from the solution in different forms, such as powder, fiber and sponges [30]. The molecular weight (Mw), degree of deacetylation (DA), ionic concentration, pH, the nature of the acid and the distribution of acetyl groups along with the main chain essentially influence the solubility of chitosan. Being a cationic polymer, chitosan displays instability in media with variable pH and ionic strength. This biopolymer has a variety of unique functional characteristics, such as biodegradability, biocompatibility, nontoxicity, antibacterial and antifungal properties. Its biological properties depend on factors such as the DA, Mw, polymerization, viscosity and dissociation constant. It has versatile mechanical properties, which have led to its enhancement of use in different applications such as encapsulation technology and controlled release coatings [31].

The most useful property of chitosan in agriculture is that it can act as a trigger in plant defense against pathogenic microorganisms. In addition, chitosan shows broad-spectrum antimicrobial effects against bacteria, fungi and viruses. Generally, chitosan is more effective against fungi than bacteria and it often exhibits higher inhibition effects on Gram-positive bacteria than Gram-negative bacteria, possibly because Gram-negative bacteria have an outer membrane structure in the cell wall affecting the cellular entry of chitosan [32]. Chitosan is widely used in agriculture in pre- and post-harvest treatments of crops to control microbial infections [18]. Chitosan-induced inhibition was observed in studies focusing on assessment of mycelial growth, sporulation, spore viability and germination and the production of fungal virulence factors. Chitosan has also been applied as a sole ingredient or composite with other elements especially with metals particles for enhanced anti-fungal effects [33]. For example, silver nanoparticles were incorporated into chitosan and tested the nanocomposite formulation as an anti-fungal agent against *Rhizoctonia solani*, *Aspergillus flavus* and *Alternaria alternata* isolated from chickpea seeds. Importantly from economical point of view, chitosan affects germination and hyphal morphology fungal of pathogens threatening harvested crops (e.g., *Rhizopus stolonifer* and *Botrytis cinerea*) [34]. This polymer also inhibits the growth of many other plant pathogenic and mycoparasitic fungi (such as *Colletotrichum* spp., *Alternaria* spp. or *Trichoderma* spp.). Sensitive fungi show energy-dependent plasma membrane permeabilization by chitosan [35]. As a broad-spectrum fungicide, chitosan has been shown to be effective against several fungal plant pathogens. It can effectively inhibit the development of phytopathogenic fungi at different life-cycle stages. Chitosan has been shown to inhibit infections caused by fungi such as *B. cinerea* or *F. oxysporum* f. sp. *radicis-lycopersici* [36]. The antifungal activity of chitosan on plant depends on the type, concentration and test organism. For example, when effect of two types of chitosan (92.1 kDa and 357.3 kDa) was tested on *Penicillium italicum*, at a concentration of 0.1%—chitosan of lower Mw was more effective in inhibiting fungal growth, while at a concentration of 0.2%—chitosan of higher Mw showed stronger antifungal activity [37].

Chitosan oligosaccharides (COS) are the degraded products prepared by chemical or enzymatic hydrolysis of chitosan or chitin derived mainly from crustacean shells. They are composed of glucosamines linked by β-1,4-glycosidic bonds [38]. The degrees of polymerization of COS are usually 2–20 [39]. In recent years, COS has received a lot of attention due to their physicochemical properties, such as high water solubility, low viscosity, biocompatibility and biodegradability. Furthermore, COS were demonstrated to have various activities in the plant protection such as inducing plant resistance to pathogens, promoting its growth and development and improving the quality and yield of plant products [40].

### 2.1. Chitosan seed Treatments

Antifungal seed treatments provide protection against seed borne or soil borne pathogenic fungi, which can significantly lower seed germination and plant emergence in the field. Chitosan in such treatments is usually applied in a form of solution for seed soaking or coating (Table 1). For example, Silva-Castro et al. [41] searched for an effective method to protect seeds and seedlings of pine trees from *Fusarium circinatum*, a dangerous pathogen threatening pine forests in Spain. They developed seed coating treatments using low and medium Mw chitosan (20 kDa and 60–130 kDa, respectively) and/or propolis ethanolic extract. They applied these treatments to the pine tree seeds inoculated with the pathogen. All coating treatment resulted in improved survival of the *Pinus sylvestris* seedlings under pathogen pressure. However, a low Mw chitosan treatment also had a positive influence on total phenolic content and antioxidant capacity of the seedlings; therefore, this seed treatment was selected as the most beneficial for protection of *P. sylvestris*.

Effectiveness of chitosan against seed borne pathogens was studied in case of *Jatropha curcas*, which is an industrial plant cultivated on many continents. Pabon-Baquero et al. [42] tested effects of chitosan on fungi (*Fusarium equiseti* and *Curvularia lunata*) isolated from ungerminated *J. curcas* seeds. Chitosan applied at different concentrations (0.5–4.0 mg mL^−1^) inhibited mycelium growth and affected sporulation and spore germination of both species in vitro. Application of chitosan on pathogen inoculated seeds reduced the infection and had no negative effect on seed germination.

Seed borne fungi (e.g., *Aspergillus niger*, *Alternaria alternata* and *Rhizopus* sp.) have a negative impact on germination of artichoke seeds leading to significant losses of this crop. Therefore, Ziani et al. [43] tested effects of chitosan seed coatings on germination of this crop. In all treatments, chitosan reduced number of fungi detected on seeds and stimulated the growth of seedlings. Chitosan with lower Mw gave better results, but it was not effective against *Rhizopus*. The combination of chitosan and commercial fungicide (tetramethylthiuram disulfide) applied at reduced concentrations resulted in a strong antifungal protection, improved germination and seedling growth.

Antifungal activity of chitosan was also tested against a soil borne pathogen, *Fusarium solani*, causing root rot in fenugreek [44]. The inhibitory effect on a mycelium growth, dry biomass, sporulation and fungal spore germination increased with the increasing concentration of chitosan (up to 2 g L^−1^) applied in vitro. The treatment of *F. solani* inoculated seeds resulted in significantly reduced infection rate of seedlings and longer radicle lengths. When tested in pot and field experiments, chitosan application on seeds reduced severity of root rot disease and increased yield. Moreover, it also resulted in stimulation of plant defense mechanisms because increased activity of chitinase and glucanase enzymes was observed in chitosan treated fenugreek plants.

A poor germination of pepper seedlings in wet and cold soil became a motivation to develop a seed treatment for this crop. Chitosan solutions (0.01–0.5%) were used to soak the pepper seeds and then germination parameters were assessed in two different temperature conditions. These treatments accelerated germination at 25 °C and improved seedling emergence in the cold test by 29%. Moreover, they increased activity of chitinase and glucanase in chitosan treated seed/seedlings compared to the untreated ones. Higher activity of these enzymes may indicate stimulation of plant defense mechanisms which may provide protection against fungal diseases [45].

Bio-based seed treatments with essential oils and plant extracts are becoming increasingly more popular due to their natural origin, faster degradation, low environmental impact and higher acceptance of the consumers avoiding chemical fungicides. Chitosan nanoparticles (NPs) with garlic essential oil were prepared by encapsulation method in order to protect the seeds of wheat, oat and barley. The new seed treatment, combining the two components, resulted in a strong antifungal activity against *Aspergillus versicolor*, *A. niger* and *Fusarium oxyporum*, comparable to the effects of standard tebuconazole treatment. Moreover, the new treatment stimulated also seed germination and seedling development what is its additional advantage apart from being an environmentally friendly alternative to chemical fungicides [46].

Attjioui et al. [47] investigated the efficacy of partially acetylated chitosan polymers and chitosan oligosaccharides (COS), applied alone and in combination, in vitro for their antifungal effect against the economically important seed-borne pathogen *F. graminearum*. The results showed that the antifungal activity of chitosan depends on its Mw. The analyzes revealed a dose–response relationship of three chitosans with the same DA (10%) and different Mw. Low Mw polymers were slightly more active than high Mw polymers or COS. However, synergistic effects of the chitosan polymer and COS were also observed on the growth of *F. graminearum*.

The combined treatment of COS and ε-poly-l-lysine had a highly inhibitory effect (inhibition rate exceeding 90%) on the destructive fungus *Botrytis cinerea* causing tomato gray mold [48]. In another study on effects of COS on the same pathogen, high fungal control efficiencies were detected and explained by that fact that COS induce plant disease resistance [49].

### 2.2. Chitosan Treatments Dedicated for Field Application

Chitosan is frequently combined with metal NPs in order to lower their toxicity (Table 2). Antifungal effects of such combination were explored by Dananjaya et al. [50] who searched for an environmentally friendly method of controlling *F. oxysporum* species complex causing infections of a broad range of plant and animals hosts. They developed chitosan NPs and chitosan-silver nanocomposites and compared their impact on the growth of *F. oxysporum* in vitro. Both solutions caused significant inhibition of fungal growth (although this effect was significantly stronger for nanocomposite with silver), and ultra-structural analysis revealed the signs of mycelium damage: higher membrane permeability, disruption of the mycelium surface and cell disintegration. These findings show that chitosan-based NPs and nanocomposites can effectively damage the pathogen and can be used in fungal control treatments. In another study, chitosan and its nanocomposites with either silver NPs, ZnO or CuO were evaluated as potential antifungal agents against *Fusarium oxysporum* f. sp. *ciceri* causing Fusarium wilt on chickpea. The strongest antifungal effects were recorded for nanocomposites of chitosan with ZnO and CuO in tests performed in vitro and in vivo; these nanocomposites showed also lower toxicity [51].

The impact of NPs containing copper and chitosan on Curvularia leaf spot disease and the growth of maize were studied by Choudhary et al. [52]. Cu-CS NPs comprehensively inhibited in vitro mycelial growth of *Curvularia lunata*. Plants treated with this formulation showed lower disease severity compared to the other studied formulations (NPs, bulk chitosan, CuSO4 and fungicide and water as control). Conclusion drawn from pot and field experiments was that application of Cu-CS NPs unquestionably controls the disease, boosts plant growth and yield.

Tomato plants may be attacked by *Pythium* spp. during early stage of growth, causing seed rot, pre-emergence damping-off, or stem rot symptoms and by *Fusarium oxysporum*, causing the most epidemic vascular wilt and root rot diseases [53]. Elsherbiny et al. [54] examined antifungal activity of chitosan nanocomposites loaded with antioxidants (vanillin and cinnamaldehyde). Samples were prepared by intercalation of chitosan into sodium montmorillonite, polyaniline and incorporation of chitosan/polyaniline/exfoliated montmorillonite. The obtained nanocomposites showed strong inhibitory effects on the linear growth of the target both pathogens even at 50 mg mL^−1^ concentration.

Saponines are complex glycosidic compounds which belong to plant secondary metabolites. Fungistatic activities of saponine-rich extracts were demonstrated by Chapagain et al. [55]. In another study, saponin was one of the substances combined in NPs with chitosan in order to enhance its antifungal properties [56]. Other NPs included chitosan, saponin, copper or chitosan combined with copper. The prepared NPs were evaluated for their effect on the growth of three phytopathogenic fungi in vitro. Among the various tested formulations, NPs comprising of chitosan and copper were found the most effective at 0.1% concentration and showed 60–90% growth inhibition of the tested fungi and a maximum (87.4%) inhibition rate on *Alternaria alternata* spore germination. Pure chitosan NPs at the same concentration showed the strongest effect on mycelial growth of *Macrophomina phaseolina*. Therefore, chitosan-based NPs with or without copper can used for plant protection in the future [56].

In another study, chitosan-pectin NPs encapsulated with carbendazim were produced. The method of ionotropic gelation was used, and the experiment focused on fungicide release in vitro and bio-efficacy. Characterization of the synthesized NPs showed that the size of the NPs encapsulated with carbendazim was 70–90 nm, the encapsulation efficiency was 99.2% and the Zeta potential was 50.2 mV. The nanoformulation showed 100% inhibition of test fungi against *Fusarium oxysporum* and *Aspergillus parasiticus*. Carbendazim nanoformulation requires less fungicide and therefore it is a more environmentally friendly method of controlling phytopathogenic fungi. This nanoformulation showed a greater efficacy at a lower concentration compared to the top commercial form of the fungicide against the target species [57].

### 2.3. Pre- and Post-Harvest Crop Protection Based on Chitosan

Chitosan represents a model plant protection biopolymer which is sustainable for control of post-harvest decay of fresh fruits and frequently used for this purpose (Table 3). One of the most important causes of harvested fruit decay is *Penicillium expansum* responsible for blue mold. Madanipour et al. [58] assessed the effect of post-harvest chitosan application in combination with licorice ethanol extract on shelf-life of apple fruits. Chitosan-licorice edible coating inhibited *P. expansum* growth and reduced post-harvest decay rate. In general, chitosan was more effective when combined with licorice extract. The results of this research support the idea that coating may be a safe alternative method to prolong shelf-life and reduce post-harvest losses of apple and maybe other fruits in storage time. In another study, infections caused by mold fungi were controlled by chitosan combined with essential oils (EOS), which are also known for their antifungal activity [59]. The effectiveness of chitosan-based films integrated with the EOS from *Mentha piperita* L. or *Mentha* × *villosa* Huds was evaluated in cherry tomato fruits. Main antimicrobial compounds present in these essential oils are rotundifolone and menthol. They belong to monoterpenes and have the ability to disorganize the membrane structure, resulting in depolarization and morphological alterations, interfering with fungal metabolism. The obtained films were edible and effectively controlled infection caused by fungi such as *Penicillium expansum*, *Botrytis cinerea*, *Rhizopus stolonifera* and *Aspergillus niger*.

Postharvest decay of table grapes is causing significant losses of the crop attributed to pathogenic fungi, such as *Botrytis cinerea*. In a process of searching for alternative to sulfur dioxide fumigation of grapes, Shen and Yang [60] developed edible coatings for these fruit using chitosan in combination with salicylic acid, which is a phytohormone promoting plant resistance. The coatings made of both ingredients induced the activities of phenylalanine ammonia lyase, chitinase, β-1, and 3-glucanase, and reduced the decay of table grapes by inhibiting the growth of *B. cinerea*. A composite coating formulation containing 1% chitosan-salicylic acid successfully decreased the respiration rate and delayed changes in weight loss, measurement of total soluble solids, titratable acidity and total phenolic content and sensory attributes of table grapes during storage. The amino group of chitosan interacts with the carbonyl group of salicylic acid to form a conjugate molecule [60]. Another eco-friendly plant protection method for the same purpose was developed by Youssef et al. [61]. They utilized chitosan nanoparticles (CS NPs), silica nanoparticles (SN NPs) and chitosan-silica nanocomposites (CS-SNs) and tested their impact on *B. cinerea* growth inhibition in vitro and in vivo, on two grape cultivars ‘Italia’ and ‘Benitaka’. In vitro tests showed that compared to control, CS NPs, SN NPs and CS-SNs reduced fungal growth by 72, 76 and 100%, respectively. After natural infection, at the end of cold storage, application of CS-SNs was also the most effective treatment; it reduced the development of gray mold by 59–83%, depending on cultivar. Since these nanocomposites had no negative effect on fruit quality, they are a promising alternative to fungicides controlling gray mold on grapes.

NPs with chitosan were used in another study aiming at protecting bell peppers from mold fungi. Gonzalez-Saucedo et al. [62] combined them with an extract obtained from leaves of nanche (*Byrsonima crassifolia*). Antifungal activity of the obtained NPs was confirmed in in vitro tests by recording up to 100% growth inhibition of *Alternaria alternata*. Edible coatings with these NPs sprayed on bell peppers before harvest reduced infections and improved storability of the crop; after storage reduced weight loss and better physicochemical features of peppers were observed.

One of the very complex edible coatings was developed for controlling green mold in harvested oranges [63]. It consists of chitosan integrated with phenolics-rich pomegranate peel extract and a biocontrol agent (*Wickerhamomyces anomalus*). The strongest effectiveness against *Penicillium digitatum* was observed in case of coating combining all of the above-mentioned components what confirmed synergistic effect of their activity.

**Table 1:** Examples of antifungal seed treatments based on carbohydrate biopolymers (chitosan, alginate or cellulose) or their derivatives.

Form of Application	Chemical Composition of the Seed Treatment Formulation *		Target Pathogen(s) (Plant Disease)	Effects of the Treatment	Ref.
	Carbohydrate Polymer or Its Derivative	Other Components			
Seed coating	CS (low and medium Mw)	propolis extract, Tween 80, Halloysite	*Fusarium circinatum* (pre- and post- emergence damping off in pine seedlings)	All coatings significantly reduced the post-emergence mortality of *Pinus sylvestris* seedlings inoculated with *F. circinatum*; coating with low Mw chitosan also had a positive influence on total phenolic content and antioxidant capacity of the seedlings.	[41]
Seed coating	CS		Fungi isolated from *Jatropha curcas* seeds: *Fusarium equiseti*, *Curvularia lunata*	Inhibited mycelium growth, sporulation and spore germination in vitro; improved germination of *J. curcas* seeds inoculated with *F. equiseti* or *C. lunata.*	[42]
Seed coating	CS	Fungicide: tetramethylthiuram disulfide; Span 80	seed borne fungi on artichoke seeds e.g., *Rhizopus* sp., *Aspergillus* sp.	Stimulated formation of an abundant root system, reduced fungal infection of seeds/seedlings, but *Rhizopus* sp. is effectively inhibited only by the fungicide.	[43]
Seed treatment	CS	HCl, NaOH	*Fusarium solani* (root rot disease on fenugreek)	Significantly reduced growth, sporulation, dried biomass and spore germination of *F. solani*	[44]
Seed treatment	CS	Fungicide: Benomyl [methyl 1-(butylcarbamoyl) -2-benzimidazole], acetic acid	Soil borne pathogens threatening pepper seeds	Improved the germination at 25 °C, higher emergence in cold test, increased activity of chitinase and glucanase in chitosan-treated seeds compared to the untreated ones.	[45]
Seed treatment	CS	CS-Garlic EO NPs, sodium tripolyphosphate	*Fusarium oxyporum*, *Aspergillus versicolor*, *Aspergillus niger* (Fusarium head blight, wilt and root rot on cereals)	Synergistic effect of CS NPs and garlic EO resulting in a strong antifungal activity; stimulated germination and seedling growth.	[46]
-	COS	-	*Fusarium graminearum* (crown/root rot, Fusarium head blight in cereals)	Antifungal effects against *F. graminearum*, affected conidia germination and caused ultrastructural modifications of fungi	[47]
Spray	COS	ε-poly-l-lysine	*Botrytis cinerea* (tomato gray mold)	Strong antifungal, synergistic effect of application of two bio-fungicides in combination, inhibition rate of *B. cinerea* > 90% in vitro, effective protection of the plants in vivo	[48]
Spray, seed treatment	COS	TEMPO, NaBr, NaOCl, NaOH, HCl	*B. cinerea*	Effective control *B. cinerea* on tomatoes and better antifungal activity, significant growth stimulation of cucumber seedlings	[49]
Seed treatment	AG	*Bacillus subtilis*, bentonite, starch and titanium dioxide NPs	*Rhizoctonia solani* (seed decay and damping-off of bean seedlings)	The application of encapsulated *B. subtilis* on inoculated bean seeds provided stronger disease inhibition compared to free bacteria. It also increased the parameters of vegetative growth of bean plants.	[64]
Seed treatment	AG	Three strains of *Streptomyces* spp.	*Ganoderma boninense* (Basal stem rot disease on oil palms)	*S. palmae* CMU-AB204T strain exhibited the strong antifungal activity in vitro. It was also the most effective in suppressing the disease on oil palm seedlings in vivo.	[65]
Seed treatment	AG	Ag NPs, aldehyde	*Colletotrichum lagenarium*, *Sclerotinia sclerotiorum*, *C. gloeosporioides*, *F. solani*, *Sphaeropsidales*, *R. solani*	Nanopesticide with a broad-spectrum antifungal activity in vitro. No negative effects on seed germination were detected.	[66]
Seed treatment	AG	Silica NPs, EOS from: *Cymbopogon citratus*, *Syzygium aromaticum*	*Gaeumannomyces* *graminis* var. *tritici*	The rate of disease control was >20% higher than in control when tested on wheat	[67]
Seed coating	EC, HEC, MC	sodium lignosulfonate, lauryl sulfate	storage fungi e.g., *Aspergillus niger* (seed deterioration in storage)	After few months of storage: lower moisture content of the seeds, higher germination percentage, higher emergence in the field and lower fungal infestation.	[68]
Seed treatment	CMC	Biocontrol microorganisms: *Bacillus cereus*, *Trichoderma harzianum*	*F. graminearum* (cereal damping-off complex)	Reduced disease severity after seed treatment with biocontrol in controlled conditions.	[69]
Seed coating	CMC	Fungicides: difenoconazole, fludioxonil FSC, LAE-9, NNO, polyacrylamide, ethylene glycol, gelatin, pigment	*Rhizoctonia cerealis* (Sharp eyespot of wheat)	Reduced severity sharp eyespot disease in the field.	[70]
Electrospun seed coating	CDA nanofibers	Pesticides (abamectin, fluopyram) acetone, dimethyl acetamid	soil borne fungi e.g., *Alternatia* spp. (soil borne diseases of soybean)	Laboratory tests showed: slow release of pesticides in water environment and growth inhibition of *A. lineariae* by fluopyram released from nanofibers.	[71]
Electrospun seed coating	CA nanofibers	Cu^2+^, gelatin surfactant (Tween80), acetic acid	*Fusarium oxysporum*	Promoted seed germination in diseased media, increased seedling biomass.	[72]

* Abbreviations: CS—Chitosan, Mw—molecular weight, AG—Alginate, COS—Chitosan oligosaccharides, EOS—Essential oils, CMC—Carboxymethyl cellulose, MC—Methyl cellulose, EC—Ethyl cellulose, HEC—Hydroxyethyl cellulose, CA—Cellulose acetate, CDA—Cellulose diacetate, TEMPO-2,2,6,6-Tetramethylpiperidine-1-oxyl, NPs—Nanoparticles.

## 3. Alginate

Alginates are naturally occurring polymers showing low toxicity, good biocompatibility and biodegradability [73]. Excellent gelling and thickening properties as well as low production cost and good availability make them easy to develop and use. One of the most important and commonly utilized features of these polysaccharides is the ability to undergo ionotropic gelation, which is gel formation process occurring upon the contact with divalent cations. The gelation mechanisms of alginate very often act according to the model, known as egg-box, where the Ca-binding sites show a mirror symmetric conformation [74].

Alginates are obtained by extracting alginic acid in alkaline solutions from brown algae. Alginic acid consists of β-D-mannuronic (M) and α-L-guluronic (G) residues linked by a β-1,4-glycosidic bond. The ratio of the participation of M and G and their distribution in the chain determines their gelation. For example, alginates derived from *Laminaria hyperbore* are characterized by an enrichment of the density of guluronic fragments (G) compared to alginates derived from *Acophyllum nodosum* or *Laminaria japonica*. Alginates have found application in many industrial sectors such as biomedical, pharmaceutical, tissue engineering and agriculture [75,76].

These polysaccharides can be combined with a wide range of substances, such as phytohormones, amino acids, fatty acids and microelements, and used in agriculture as organic fertilizers, delivery systems, seed treatments and edible coating films for vegetable and fruits. For example, alginates with chitosan and other substances were used in soybean seed coatings [77,78]. Alginate coatings have also been reported to be good oxygen barriers and to reduce the natural microflora counts [79]. Moreover, they stimulate the growth of aerial parts and the root system of plants and increase their resistance to pathogens [80].

Alginates were commonly used for encapsulation of microorganisms [81]. Biofertilizers, namely *Rhizobium* (Gram -), and biocontrol agents, such as *Pseudomonas* (Gram -) and *Trichoderma*, have been well established in the field of agricultural practices for many decades [82]. The use of conventional liquid or solid formulations in agricultural areas causes many problems, mainly due to the poor viability of the microorganisms during storage and field application. Encapsulation technology helps to overcome these problems. This form of immobilization of microorganisms results in extended shelf-life and controlled release of microorganisms from the preparations, which increases the effectiveness of their use in the field [73,83].

Alginate oligosaccharides (AOS), which are degradation products of alginate, show more attractive biological activity due to their low molecular weight [84,85]. AOS exhibit excellent potential for agricultural applications because they promote plant growth, alleviate growth inhibition under abiotic stress, induce defense responses in plant and extend a shelf-life of harvested crops [86,87].

### 3.1. Alginate Seed Treatments

Sodium alginate is often utilized in encapsulation process because of its excellent features, such as appropriate morphology, fiber size, porosity, degradation and swelling ratio [88]. This polymer was used to develop an innovative encapsulation system for delivery of biocontrol bacteria *Bacillus subtilis* (Vru1). The formulation contained also bentonite, starch and titanium dioxide NPs [64]. The purpose of encapsulation was to protect the bacteria from harmful environmental conditions and strengthen their survival rate so that they could provide an effective control of *Rhizoctonia solani* infection on bean plants. Greenhouse tests of different variants of control treatments on fungal inoculated bean seeds showed that encapsulation enhanced antifungal effects of *B. subtilis*, because it led to stronger disease inhibition compared to treatment with free bacteria. The application of encapsulated *B. subtilis* has also significantly increased the growth of bean plants. Therefore, the developed nanocapsules with biocontrol bacteria are a potential alternative for sustainable agriculture.

Alginate was also used in a new method to control oil palm disease, which was meant to be an alternative to pesticide treatments [65]. The oil palm trees are frequently damaged by the fungal infection (basal stem rot disease) caused by *Ganoderma boninense*. The selected three strains of biocontrol bacteria *Streptomyces* spp. exhibited the strongest degree of anti-*G. boninense* activity in vitro. Therefore, the effectiveness of these microorganisms on suppressing the disease symptoms was tested in vivo on oil palm seedlings using spore immobilized in alginate beads. Formulation with *S. palmae* CMU-AB204T strain resulted in the lowest disease severity and the highest degree of plant vigor. Therefore, this strain can be used as biocontrol agent protecting palm trees from basal stem rot disease [65].

Xiang et al. [66] developed a new high-performance nanopesticide with a broad-spectrum antifungal activity. It is comprised of silver NPs synthesized from aldehyde modified sodium alginate (SA-AgNPs). The synthesized SA-AgNPs showed a strong antifungal activity against the following pathogens: *Colletotrichum lagenarium*, *Sclerotinia sclerotiorum*, *C. gloeosporioides*, *Fusarium solani*, Sphaeropsidales and *Rhizoctonia solani*. This activity was associated with the impact of SA-AgNPs on fungal membrane permeability, soluble protein synthesis, destruction of DNA structure and inhibition of its replication. The new SA-AgNPs showed no inhibition of seed germination hence their phytotoxicity was excluded.

Essential oils (EOS) obtained from *Cymbopogon citratus* and *Syzygium aromaticum* were used against *Gaeumannomyces graminis* var. *tritici*, an aggressive pathogen causing a take-all disease of wheat. To provide a controlled delivery of the EOS, they were encapsulated into mesoporous silica NPs and then sodium alginate was used to keep these NPs around the seeds. Effects of this formulation were compared to the effects of pure EOS both in vitro and in vivo. Encapsulating of EOS successfully increased their stability in the environment, allowed their controlled release and reduced fungicidal dose. Results confirmed compatibility of alginate with natural fungicidal compounds and its positive impact on effectiveness of the whole formulation [67].

### 3.2. Alginate Treatments Dedicated for Field Application

Alginates were used in slow-release systems with Bosphorus (formerly nicobiphene) which is a broad-spectrum fungicide that is safe for plants. It can inhibit the respiration of fungi by binding to the enzyme succinate dehydrogenase in fungal mitochondria. To control the fungicide release rate, a slow-release boscalid composition for the treatment of gray mold was patented. The authors used bentonite, cationic surfactant and sodium alginate solution for dispersion as agent for controlling cucumber Botrytis [89]. In another study, agar and alginate beads containing thiram, were produced in order to slow down the release of active fungicide in vitro and in soil. The amount of active ingredient available for leaching and volatilization was decreased from the beads and the availability of the fungicide in the soil was prolonged. The release of thiram decreased with increasing alginate concentration in the feed from 1% to 2.5% (w/v), which explained the progressive shrinkage of the alginate spheres, which in turn led to an increase in Ca^2+^ alginate cross-link density and a decrease the size of the pores. A slower release of thiram in soil compared to in vitro conditions what was explained by the occlusion of the ball surface by soil particles slowing diffusion, and also by dissolved soil water, which can also delay pesticide displacement [90].

The alginate oligosaccharides (AOS) combined with *Meyerozim guilliermondii* have been studied as a possible physiological biocontrol against *Penicillium expansum* infection of pears. Blue mold caused by *P. expansum* was significantly inhibited by the developed formulation in concentration of 5 g L^−1^, while it did not affect the growth and reproduction of *M. guilliermondii* in vitro or in vivo on pears [91].

### 3.3. Pre- and Post-Harvest Crop Protection Based on Alginate

Coating films on fruits and vegetables with preservative compounds slow down ripening and senescence due to formation of a modified atmosphere around the fruits and vegetables thereby reducing the respiration rate [92]. These coatings are made of edible material which provides a moisture barrier and prevents solute movement from the food [93]. They can be made of biodegradable raw material, such as polysaccharides, and serve as a primary packaging which is directly in contact with the fruit, wrapping it to form a gas and moisture barrier, improving the mechanical property, reducing the microbial load, keeping the sensory properties intact while prolonging the shelf-life [94]. Fruit and vegetable coatings based on alginates can exhibit all these qualities and they may be combined with diverse active ingredients (Table 3). For example, Xu et al. [95] described the inclusion of cyclolipopeptides (CL) from *Bacillus subtilis* in the production of an easily removable alginate coating for preserving blueberries. The obtained CL-alginate coatings provided strong antifungal properties and kept blueberries fresh during 20 days of cold storage. Fungal contamination of the coated fruits was reduced to 2.5 × 103 cfu g^−1^ and it was at least 10 times lower compared to uncoated control. Moreover, the coating resulted in higher firmness, reduced respiratory rate and reduced weight loss in the stored blueberries.

Essential oils (EOS) are hydrophobic concentrated liquids derived from aromatic plants. They contain a multitude of bioactive compounds such as antimicrobial and antioxidant, and can be used as preservative for fruits. Therefore, they may be used to maintain the quality and shelf-life of fresh-cut fruits. Fresh-cut papaya pieces were treated with alginate based edible coatings containing thyme and oregano EOS, which constituted the lipid component of the coating. Increasing concentration of essential oil resulted in extended shelf-life and a higher moisture retention capacity of the samples. However, the strong smell of essential oils, caused a negative reaction from the sensory panel. Nevertheless, positive effects of the coatings include reduced weight loss, retarded pH changes, reduced respiration rate and delayed senescence. The reduced microbial growth may be due to the incorporation of essential oil as well as due to the modified atmosphere created by the coating [96]. Similar alginate-based coatings combined with EOS were shown to extend self-life of fruits and reduce the counts of microorganisms in case of raspberries, fresh-cut apples and pineapples [97,98,99] (Table 3). Another study described the use of an alginate/vanillin combination to improve the quality and safety of table grapes. The pre-harvest spray and post-harvest fruit coating was applied to three grape varieties. Alginate treatments effectively prevented weight loss and firmness loss of the fruit. Moreover, alginate/vanillin coating provided a significant reduction in yeast-mold growth. In addition, it maintained the nutritional and sensory quality of grapes, preserved functional properties (such as phenolic content and antioxidant activity) and extended their shelf-life by diminishing fungal decay [100].

Alginate-based coatings of fruits can be enriched with plant extracts in order to enhance their antifungal properties. For example, rhubarb (*Rheum rhaponicum* L.) extract, known for its antifungal and antiseptic activity, was combined with sodium alginate in a coating for peach preservation [101]. Alginate coatings (1% sodium alginate) reduced weight loss, firmness loss and respiratory rate and resulted in higher nutritional value of the stored coated fruits compared to uncoated control fruits. Moreover, a significantly lower decay index was recorded for alginate-coated fruit previously inoculated with *Penicillium expansum*, which was explained by reduced gas exchange inhibiting growth of molds which are aerobic. However, all of the above-mentioned positive effects of alginate coating were significantly enhanced by addition of rhubarb extract, therefore a coating combining both components was recommended as a treatment prolonging shelf-life of peach fruits.

The addition of nanomaterials can also enhance the antifungal activity of the alginate coatings. Jiang et al. [102] reported the efficacy of a composite alginate/nano-Ag coating in reducing the counts of different groups of microorganisms on coated shiitake mushrooms after the cold storage for 16 days compared with the uncoated control.

The addition of ZnO nanoparticles in different concentrations to sodium alginate-coatings resulted in the enhanced shelf-life of cold stored strawberries by preventing the loss of weight, sensory attributes and the reduction in ascorbic acid, total phenols and total anthocyanins content. The nano-coating, which was the most effective in reducing the counts of yeast, molds and aerobic bacteria in cold stored strawberries, comprised 1.5% sodium alginate and ZnO nanoparticles at the concentration of 1.25 g L^−1^ [103].

Zhuo et al. [104] demonstrated that AOS treatment improved resistance to post-harvest decay and quality in kiwifruit (*Actinidia deliciosa* cv. ‘Bruno’). The results showed that in vitro AOS did not inhibit the growth of *Botrytis cinerea*, which is the causal agent of gray mold in kiwifruit, but they reduced the incidence of gray mold and diameter of lesions of kiwifruit during storage.

**Table 2:** Examples of antifungal treatments dedicated for field application and based on carbohydrate biopolymers (chitosan, alginate or cellulose) or their derivatives.

Form of Application	Chemical Composition of the Seed Treatment Formulation *		Target Pathogen(s) (Plant Disease)	Effects of the Treatment	Ref.
	Carbohydrate Polymer or Its Derivative	Other Components			
-	CS	CS NPs, CS-Ag NCs, AgNO_3_, TPP, NaOH, Na_5_P_3_O_10_	*Fusarium* *oxysporum*	Reduced fungal growth in vitro, morphological and ultrastructural changes in of the mycelium	[50]
Soil application	CS	CS-Ag CS-CuO, CS-ZnO	*F. oxysporum* f. sp. *Ciceri* (Wilt disease of chickpea)	Nanocomposites of chitosan combined with CuO or ZnO provided the most effective protection against wilt disease and promoted growth of chickpea plants	[51]
Seed treatment, foliar application	CS	CS-Cu NPs	*Curvularia lunata*, (Curvularia leaf spot disease of maize)	Lower disease severity observed in maize in pot and field experiments, plant growth stimulation.	[52]
Seedling treatment	CS	vanilin, cinnamaldehyde, polyaniline, sodium montmorillonite	*Pythium* spp. *Fusarium oxysporum* (root rot, pre-emergence damping off in tomato plants)	Strong inhibitory effect on the linear growth of both target pathogens, reduced disease incidence under greenhouse conditions	[54]
-	CS	CS-Saponin NPs, CS-Cu NPs, TPP	*Alternaria alternata*, *Macrophomina phaseolina*, *Rhizoctonia solani*,	Compared to CS-Saponin NPs, CS-Cu NPs were more effective and caused fungal growth inhibition in vitro of 89.5%, 63.0% and 60.1% in case of *A. alternate*, *M. phaseolina* and *R. solani*, respectively.	[56]
Encapsulation	CS	CS-pectin NPs, fungicide: carbendazim	*F. oxysporum*, *Aspergillus parasiticus*	100% inhibition of tested fungi. Carbendazim nanoformulation showed greater efficacy at a lower concentration compared to the top carbendazim and commercial form against target species	[57]
Foliar spray	CS	CS-Cu NPs, CuSO_4_, fungicide: Bavistin	*Curvularia lunata* (Curvularia leaf spot in maize)	Significant defense response and control of the disease in maize.	[52]
Encapsulation	AG	Fungicide: Bosphorus -(formerly nicobiphene); bentonite	*Botrytis. cinerea* (gray mold on cucumber)	Broad-spectrum fungicide inhibits the respiration of fungi by binding to the enzyme succinate dehydrogenase in fungal mitochondria.	[89]
Encapsulation	AG beads	Fungicide: thiram	various fungi	Slower release the active fungicide in vitro and in the soil.	[90]
Spray	AOS	*Meyerozyma* *guilliermondii*	*Penicillium* *Expansum* (blue mold on pears)	The results showed that AOS (5 g/L) combined with *M. guilliermondii* significantly reduced blue mold decay incidence and lesion diameter in pears.	[91]
Encapsultion	EC	Fungicide: fluazinam; gum arabic, emulsifier	*B cinerea* (gray mold on cucumber)	In in vitro tests: stronger inhibitory effect on *B. cinerea.* In the field experiment: slower degradation after spraying plants and no phytotoxic effects on plants in case of encapsulated fungicide compared to fungicide suspension.	[105]
Nano- carriers	fatty acid cellulose ester	Fugicides: captan, pyraclostrobin	*Neonectria ditissima*, *Phaeoacremonium minimum* (Apple Canker and Esca disease of grapevine)	In in vitro tests: pesticide release in contact with cellulolytic fungi and fungal growth inhibition	[106]
Nano- carriers	HPC	Fungicide (pyraclostrobin); silica NPs	*Magnaporthe oryzae* (rice blast)	Fungicide release induced either by low pH or cellulase. Prolonged photostability and reduced cytotoxicity of the fungicide delivered in nanocarriers compared to commercial formulations.	[107]
Micro-spheres	Copolymer: CS, CMC	EOS: citral	*B. cinerea* (gray mold in solanaceous crops)	Antifungal activity in vitro and reduced disease incidence in tomato tested in vivo	[108]
Electrospun memebrane	CA	5-chloro-8-hydroxyquinolinol, polyethylene glycol, acetone	*Phaeomoniella* *chlamydospora*, *Phaeoacremonium aleophilum* (Esca on grapevine)	Membranes prevent fungal spore penetration of plant tissues wounded by pruning procedure	[109]

* Abbreviations: CS -Chitosan, AG—Alginate, AOS—Alginate oligosaccharides, EOS—Essential oils, CMC—Carboxymethyl cellulose, HPC—Hydroxypropyl cellulose, CA—Cellulose acetate, NPs—Nanoparticles.

## 4. Cellulose

Cellulose is the most abundant carbohydrate biopolymer in nature. It is produced by plants in a photosynthesis process and it plays an important, structural role in these organisms. Cellulose can be obtained from plant material, such as wood, cotton, flax, water plants, grasses, agricultural residues and from bacteria belonging to few genera. However, the main sources of this polymer for commercial production are wood and cotton [110,111].

Cellulose consists of long chains of beta (1-4)-glycosidically linked glucose units. It is insoluble in water and common organic solvents. Cellulose can be converted into a variety of derivatives with different functionalities through etherification or esterification [111]. Cellulose ethers commonly used in agriculture include: methyl cellulose (ME), ethyl cellulose (EC), hydroxyethyl cellulose (HEC), hydroxypropyl cellulose (HPC), hydroxypropyl methylcellulose (HPMC) and carboxymethyl cellulose (CMC). These derivatives are characterized by higher solubility in water and/or organic solvents compared to cellulose. Their properties allow for using them as thickeners, binders or coating agents in formulations applied as foliar sprays, seed treatments or edible films on food products.

Among esters, the most important compounds include cellulose acetates (cellulose acetate—CA; cellulose diacetate—CDA) which are tasteless, nontoxic, relatively stable in storage, insoluble in water and easily biodegradable [111], which make them suitable for use in organic agriculture and food production. Moreover, they are suitable substrates for production of electrospun nanofibers, which can be used in biodegradable membranes and coatings of plant material for the targeted delivery of agrochemicals [112].

Cellulose derivatives are commonly used in plant protection applications as binders and carriers of active ingredients and biocontrol agents. However, they can also form biodegradable membranes and coatings of plant material, which can form a protective physical barrier from the environment.

### 4.1. Cellulose Seed Treatments

Seed coating procedure requires using liquid substances which will bind solid materials and active ingredients to the seed surface. These liquids, called binders, are responsible for integrity and durability of the coating during its application and after drying [113]. Cellulose ethers are relatively frequently used for this purpose. Pedrini et al. [114] reviewed 127 publications on seed coating methods and found that at least one of the five cellulose ethers (MC, EC, HEC, HPC and CMC) was used in approximately 20% of non-commercial seed coatings with a known composition.

Polymeric coatings without active ingredients can act as physical barrier preventing moisture from entering the seeds during storage thereby preventing development of fungi and deterioration of the seeds. Such seed treatments are particularly important in countries where seeds are stored in the conditions of a high temperature and a high moisture, which are highly detrimental for the stored seeds. Kumar et al. [68] tested effects of various polymer seed coatings on storability of soybean seeds. Some of the coatings included in their study consisted of MC, EC or HEC. After six months of storage, coated seeds showed lower moisture content and higher emergence in the field compared to uncoated control seeds. Coating with cellulose derivatives resulted also in lower percentage of seeds infected with storage fungi, such as *Alternaria alternata*, *Aspergillus niger*, *Curvularia lunata*, *Dreschlera halodes*, *Fusarium moniliformae*, *Cladosporium* spp. and *Penicilium* spp.

CMC can be used as base for formulations containing microorganisms. For example, Viji et al. [115] used this cellulose ether in formulations containing biocontrol agent-bacterial strains of *Pseudomonas aeruginosa*. Foliar sprays using these formulations effectively reduced the gray leaf spot disease in perennial ryegrass. In another study, the same polymer was combined with biocontrol microorganisms in seed treatment formulation protecting wheat against *Fusarium graminearum* [69].

CMC is often used in seed coatings containing fungicides. Such coatings consist usually of several components. Ren et al. [70] optimized the coating formulation for protection of germinating wheat seeds from soil borne diseases. They tested effects of few polymers on the emergence and growth of wheat seedlings and concluded that best results are obtained for CMC combined with polyacrylamide. Therefore, these two polymers were selected as binders for the seed coating delivering fungicides: difenoconazole and fludioxonil FSC designed for control of sharp eyespot disease in wheat.

Pesticides delivered in the seed coating protect germinating seeds only for a short time if they are rapidly released to the environment. Many soil-borne pathogens may threaten seeds, seedlings and young plants; therefore, a more sustained release of plant protection agents from the coating would be desirable. A choice of polymer for the coating seems to be an important decision in this context. Farias et al. [71] chose CDA for developing a new nanofiber coating for soybean seeds. In contrast to other electrospinnable polymers such as polyvinylpyrrolidone (PVP) and polyvinyl alcohol (PVA), CDA is hydrophobic in nature. Laboratory tests of the new nanofibers with abamectin or fluopyram, showed that only 5.5–25% of the total content of these pesticides was released during 2 weeks of soaking in the water. Nanofibers spun directly on the soybean seeds had no detrimental effects on seed germination. Moreover, in vitro tests with fluopyram loaded nanofibers showed also that the released fungicide cased a significant growth inhibition of *Alternaria lineariae*.

Recently, Xu et al. [72] used a method of electrospinning two biopolymers (CA and gelatin) to produce a copper (Cu^2+^) loaded nanofiber seed coating for protection against soil borne diseases. They avoided using toxic organic solvents (such as acetone or dimethyl acetamide), therefore their solution is more environmentally friendly. Interestingly, they showed that the kinetics of Cu^2+^ release from nanofibers can be altered by using different proportions of two biopolymers and adding a surfactant. The effectiveness of new seed treatments was tested in greenhouse experiments in which coated seeds of lettuce and tomato were germinated in *Fusarium oxysporum* infected media. The nanofiber coating clearly improved germination rate and plant growth under pathogen pressure.

### 4.2. Cellulose Treatments Dedicated for Field Application

CMC and EC were used as controlled release matrices for delivery of insecticides and herbicides [116]. However, recent publication of Liu et al. [105] showed that EC microcapsules with fungicide can be effectively used as plant protection against *Botrytis cinerea* causing gray mold (Table 2). This form of delivery of plant protection agent assures prolonged release and slower degradation in the environment. Moreover, encapsulation of the fungicides reduces the toxic effects on cucumber plants treated in the field.

Encapsulation of fungicides using derivatives of cellulose has one more important advantage. Many fungal pathogens of plants produce cellulase—an enzyme degrading cellulose. Therefore, nanocarriers with fungicides made of this polysaccharide will quickly disintegrate in contact with such fungi and release their cargo where it is the most needed. Machado et al. [106] produced such nanocarriers using fatty acid cellulose ester and fungicides for plant protection against severe trunk diseases of apple trees and grapevine caused by cellulase producing fungi *Neonectria ditissima* and *Phaeoacremonium minimum*. They showed in laboratory tests that the growth of both pathogens is greatly inhibited by fungicides provided in nanocarriers. In contrast, in case of fungus which is unable to produce this enzyme (*Cylindrocladium buxicola*), the effect on inhibition of the fungal growth was much smaller, because it depended only on diffusion of the fungicide from the nanocarriers.

Fungal infection exposes plant cell not only to cell wall degrading enzymes such as cellulase but also to lower pH. Therefore, Gao et al. [107] constructed nanocarriers using HPC and hollow mesoporous silica nanoparticles which release fungicide in response to either of the two stimuli.

Plant protection products comprised of nanocarriers can deliver not only fungicides but also antimicrobial substances of natural origin, such as plant essential oils. Ma et al. [108] demonstrated that hydrogel microspheres made of chitosan and CMC can be loaded with citral, which increases bioavailability of this highly volatile and unstable compound. The obtained microspheres with citral showed antibacterial activity in vitro and antifungal properties in vivo in tomato plants. They reduced incidence of the disease caused by *Botrytis cinerea*.

Another form of plant protection application was developed for grapevine protection against fungi causing esca, a damaging disease caused mainly by two species of fungi: *Phaeomoniella chlamydospora* and *Phaeoacremonium aleophilum*. In order to prevent fungal infections of the wounds formed during pruning procedure, Spasova et al. [109] produced a protective antifungal membranes using electrospinning technology. Membranes build using CA or CA and polyethylene glycol (PEG) were combined with antifungal agent: 5-chloro-8-hydroxyquinolinol. Laboratory tests of both types of membranes showed a quicker release of the pesticide from CA/PEG membrane, what was explained by a higher wettability of this material. Antifungal effect of the obtained membranes was confirmed in vitro by showing the growth inhibition of *P. chlamydospora* and *P. aleophilum*.

### 4.3. Post-Harvest Crop Protection Based on Cellulose

Cellulose ethers such as MC, CMC, HPC and HPMC are widely produced and used in edible coatings of various fruits and vegetables. They bind the coating to the surface of the product, provide moisture and create a barrier for gas exchange [26]. Out of the above-mentioned ethers, CMC is the most important for industry and commonly used in food production, also in edible coatings of fruit and vegetables. Antifungal effects of CMC coatings can be inferred from studies which test effects of coating on decay and quality of fruits after storage. For example, Kumar et al. [117] coated guava fruit with various CMC solutions (0–2.0 g L^−1^). After 12 days of storage at ambient temperature, coating with CMC at the intermediate concentration (1.5 g L^−1^) resulted in the lowest percentage of decayed fruit and best fruit quality compared to other coating treatments. In another study, Baswal et al. [118] compared effects of several coatings consisting of CMC, chitosan or beeswax on decay and quality of mandarin fruits after 75 days of cold storage. The best protection was provided by CMC coating (2.0 g L^−1^), which retained its integrity during the whole storage period. It was the most effective in maintaining fruit quality parameters, while reducing fruit decay and activity of cell wall degrading enzymes.

Next generation of edible coatings consist of polymeric matrix combined with functional and bioactive compounds which enhance the quality of the coated product and bring additional benefits to the health of the consumers [26]. For example, CMC was combined with probiotic bacteria *Lactobacillus plantarum* in edible coating created to extend a shelf-life of strawberries [119]. The bacteria remained viable on the surface of coated fruit for 15 days of cold storage and they helped to reduce the growth of yeast and molds probably due to competitive interaction with these microorganisms. The coating treatments with higher amounts of *L. plantarum* (5.76–9.80 × 10^13^ cfu mL^−1^) were the most effective in reducing fruit decay and the counts yeast and molds compared to uncoated control. CMC coating without bacteria also resulted in a reduction in fruit decay and the counts of detrimental microorganisms but its effects were intermediate compared to control and the above-mentioned *L. plantarum* coatings.

Antifungal properties of the polysaccharide coatings can be also enhanced by addition of plant extracts. It was shown in in vitro tests that extract of *Impatiens balsamina* L. stems inhibited growth of *Penicillium* molds responsible for postharvest infections of citrus fruits. Therefore, Chen et al. [120] added this extract to edible coatings of tangerine fruits based on CMC. The complex coating that was obtained included also additional substances which functioned as antioxidant, plasticizer, moisturizer, and antiseptic. Further analyses involved comparing effects of three treatments including uncoated control, CMC coating without additional substances and the complex coating described above. After 100 days of cold storage, the lowest decay rate and weight loss were recorded for fruits treated with the complex coatings. These measures for fruits coated only with CMC showed intermediate values. The complex coating resulted in the highest nutritional quality of the fruits (Table 3). Moreover, antioxidant and defense-related enzymes reached the highest activities for this treatment.

Tesfay et al. [121] isolated pathogenic three fungi from avocado fruits, namely *Colletotrichum gloeosporioides*, *Alternaria alternata* and *Lasiodiplodia theobromae*, and showed in vitro that their growth can be inhibited by extracts obtained from moringa (*Moringa oleifera* Lam.) plants or seeds. Therefore, they added these extracts to CMC to obtain antifungal coatings for avocado fruits. The new coatings improved storability of avocado fruits and their antifungal properties were confirmed in vivo. Inoculation of coated and uncoated fruits with *C. gloeosporioides* and *A. alternata* showed that the coating significantly reduced the disease incidence and severity. Therefore, the new coating treatments are suitable as organic postharvest treatment for avocado fruit.

Edible coatings based on HPMC were used for coating fruit and vegetables, but they were frequently combined with preservatives classified by EU regulations as food additives which are generally recognized as safe. Valencia-Chamorro et al. [122] developed such a coating for cold stored oranges and tested its antifungal properties in fruit which was first inoculated with *P. digitatum* or *P. italicum* and then coated with HPMC in combination with one or two preservatives and hydrophobic components. After cold storage, the coating had no negative effect on the fruit quality and its antifungal properties were confirmed. The most effective coating, containing potassium sorbate and sodium propionate, controlled development of green and blue mold on the inoculated fruit. A similar coating was developed for cherry tomatoes by Fagundes et al. [123]; however, this study aimed at controlling two important pathogens *Botrytis cinerea* and *Alternaria alternata*. A range of in vitro tests allowed to select the most effective preservative to control these fungi. HPMC-lipid coatings containing the selected preservatives were used in in vivo experiments to coat pathogen inoculated tomatoes. The coating in this case had also curative properties as the disease incidence and severity were significantly reduced although these effects were stronger in case of experiment with *A. alternata*.

**Table 3:** Examples of antifungal coatings of fruits and vegetables based on carbohydrate biopolymers (chitosan, alginate or cellulose) or their derivatives.

Fruit or Vegetable	Chemical Composition of the Coating *		Fungi Responsible for Crop Decay	Effects of the Coating	Ref.
	Carbohydrate Polymer or Its Derivative	Other Components			
Apple	CS	licorice extract	*Penicillium* *expansum*	CS-licorice coating inhibited *P. expansum* growth, reduced postharvest decay rate and weight loss of apples.	[58]
Cherry tomato	CS	EOS from *Mentha* spp.	*P. expansum*, *Botrytis cinerea*, *Rhizopus stolonifera*, *Aspergillus niger*	CS-EOS combination strongly inhibited mycelial growth and spore germination of target fungi. CS-EOS coatings reduced decay of inoculated tomato fruits and preserved quality of the stored fruit	[59]
Grapes	CS	salicylic acid, glacial acetic acid, NaOH	*B. cinerea*	Compared to pure CS coatings, coatings based on CS-salicylic acid conjugate were the most effective at promoting plant resistance, reducing fruit decay while improving their storability	[60]
Grapes	CS NPs	Silica NPs	*B. cinerea*	Compared to both types of NPs, CS-silica nanocomposites were the most effective in inhibiting *B. cinerea* growth in vitro and in vivo. No negative impact on fruit quality was observed.	[61]
Bell pepper	CS NPs	*Byrsonima* *crassifolia* extract	*Alternaria* *alternata*	CS NPs inhibited *A. alternata* growth up to 100% in vitro; when used in edible coatings in vivo they reduced the counts of microorganisms, decreased weight loss and improved quality of peppers after storage.	[62]
Orange	CS	pomegranate peel extract, *Wickerhamomyces anomalus*	*Penicillium* *digitatum*	Coatings combining CS, pomegranate peel extract and *W. anomalus* showed the strongest antifungal effect in vivo (synergistic effect of the three components confirmed)	[63]
Blueberry	AG	Cyclolipopeptides from *Bacillus subtilis*	*Aspergillus* *niger*	Compared to uncoated control, coated fruit showed >10× lower fungal contamination, reduced respiratory rate and weight loss during cold storage	[95]
Papaya	AG	Thyme and oregano EOS, Cween 80	not specified	Coatings reduced weight loss of fresh-cut fruit, retarded pH changes, reduced respiration rate thus delayed senescence	[96]
Apple	AG	EOS: lemongrass, oregano, vanillin; apple puree	*Listeria innocua*	Coatings with EOS inhibited the growth of *L. innocua* inoculated on apple pieces as well as psychrophilic aerobic bacteria, yeasts and molds	[99]
Pineapple	AG	EOS: lemongrass, glycerol, sunflower oil, ascorbic acid, citric acid	yeast and molds	Reduced weight loss, respiration rate, total counts of microorganisms, yeast and molds during storage	[97]
Raspberry	AG	EOS: citral and eugenol, ascorbic acid	yeast and molds	Improved storability, nutritional and sensory quality of fruits, growth inhibition of molds, yeasts and aerobic mesophilic microorganisms (compared to uncoated control)	[98]
Grapes	AG	vanillin, glycerol	*B. cinerea*	Maintained nutritional quality, sensory quality and extended the shelf-life of grapes, reduced growth of yeasts and molds	[100]
Peach	AG	rhubarb extract	*P. expansum*	Reduced weight loss, firmness loss, respiratory rate and higher nutritional value compared to uncoated control fruits; reduced decay index recorded for coated fruit which were previously inoculated with *P. expansum*.	[101]
Shiitake mushrooms	AG	Nano-Ag	bacteria, yeasts and molds	Enhanced shelf-life, higher physicochemical and sensory quality, reduced weight loss, lower counts of different groups of microorganisms.	[102]
Strawberry	AG	ZnO NPs	not specified	Enhanced shelf-life, reduced loss of weight, texture quality and the content of the ascorbic acid, total phenols and anthocyanins.	[103]
Kiwifruit	AOS	-	*B. cinerea*	AOS did not inhibit the growth of *B. cinerea* in vitro, but reduced the incidence of gray mold and diameter of lesions of kiwifruit during storage.	[104]
Guava	CMC	-	not specified	Reduced decay and weight loss of fruits; higher firmness; better sensory attributes; higher sugar, ascorbic acid and phenol contents; higher titratable acidity	[117]
Mandarin	CMC	-	*Penicillium* *italicum*	Best results compared to chitosan and beeswax coatings: reduced decay and weight loss of fruits; higher juice content and firmness of the fruits; lower activity of cell wall degrading enzymes; higher titratable acidity; higher ascorbic acid and carotenoids contents	[118]
Strawberry	CMC	probiotic bacteria: *Lactobacillus plantarum*, glycerol		Reduced counts of yeast and molds and reduced percentage of decayed fruits after cold storage (better results compared to control and compared to pure CMC coating); reduced weight loss, slower deterioration of ascorbic acid and phenolic compounds.	[119]
Tangerine	CMC	ethanol extract of *Impatiens balsamina* L. stems, citric acid, sucrose ester, calcium propionate, glycerol	*Penicillium* spp.	Improved results compared to pure CMC coating: lowest decay and weight loss after cold storage; highest total soluble solid, titratable acid, total sugar and ascorbic acid contents; highest activity of antioxidant and defence-related enzymes	[120]
Avocado	CMC	Moringa plant extracts	*Colletotrichum* *gloeosporioides*, *A. alternata*, *Lasiodiplodia theobromae*	Reduced decay and weight loss of the stored fruit higher firmness of the fruit; reduced ethylene production and respiration rate; confirmed antifungal effect in fungal inoculation in vivo test.	[121]
Orange	HPMC	food preservatives, shellac, beeswax, glycerol, stearic acid	*P. digitatum*, *P. italicum*	Lower incidence and severity of the disease observed on *Penicillum* sp. inoculated fruit (compared to inoculated and uncoated control). The most effective coating contained potassium sorbate and sodium propionate. Coating had no adverse effects on fruit quality.	[122]
Cherry tomato	HPMC	food preservatives	*B. cinerea*, *A. alternata*	Positive effect on the fruit quality and antifungal properties of coatings were confirmed.	[123]

* Abbreviations: CS—chitosan, AG—alginate, AOS—alginate oligosaccharides, EOS—essential oils, CMC—carboxymethyl cellulose, HPMC—hydroxypropyl methylcellulose, NPs—nanoparticles.

## 5. Advantages and Disadvantages of Carbohydrate Biopolymers Used in Plant Protection

Cellulose, chitosan and alginate are the most common carbohydrates in nature. Moreover, cellulose and chitosan can be obtained from waste material from agriculture, wood processing or crustacean shells produced by food industry. High availability is probably the most important advantage of carbohydrates considered in this review. Moreover, since these polymers are of natural origin, there are a lot of microorganisms in the environment which are able to decompose them, therefore they are highly biodegradable and suitable for organic agriculture. In addition, non-toxicity makes these biopolymers safe for the consumers and non-target organisms in the environment.

**Figure 2:**
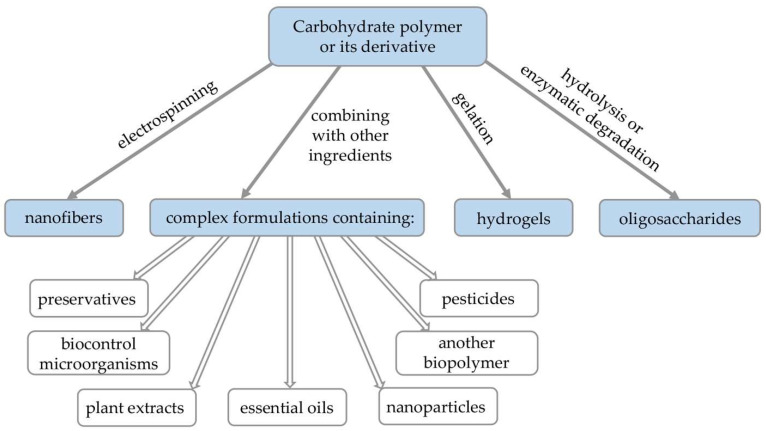
Alternative modification methods of carbohydrate polymers which can be used in the process of preparing antifungal plant protection formulations.

During preparation of plant protection formulations carbohydrate biopolymers can be subjected to a variety of different processes such as chemical modification, electrospinning, hydrolysis and gelation. Moreover, since they have the ability to interact with many hydrophobic and hydrophilic compounds, they are often combined with other ingredients in composites or complex formulations (Figure 2, Table 1, Table 2 and Table 3).

Carbohydrate biopolymers can be used in the process of encapsulation of biocontrol microorganisms in order to protect them from harmful environmental conditions and prolong their viability [64,83]. Similar solution can be used for delivering agrochemicals or volatile active ingredients (essential oils). In this case biopolymers extend activity of encapsulated compounds and ensure their slow release to the crops, thereby increasing efficiency of plant protection and reducing environmental impact of the used agrochemicals [14,16,108]. Moreover, polysaccharides in a form of hydrogels provide additional positive effect of increased water retention in the soil therefore they may help to alleviate effects of drought stress on plants [16]. Another advantage of carbohydrate biopolymers is that the products of their hydrolysis or enzymatic degradation (oligosaccharides) may act as elicitors stimulating plant defense mechanisms [19]. Chitosan, alginate and cellulose derivatives have a film forming capacity. Therefore, they are suitable as coatings for seeds or harvested fruits, which may reduce fungal growth even if they act as passive barrier [26].

Despite these advantages, carbohydrate biopolymers are not commonly utilized in plant protection due to a number of reasons. Firstly, they can be easily degraded by widespread microorganisms which is a disadvantage in the context of a short shelf-life of plant protection products based on these compounds. In order to maintain the biochemical properties and bioactivity of the biopolymer-containing product, it should also contain a preservative reducing a microbial growth. For example, a commercial plant protection product, Beta-chikol^®^, based on chitosan lactate, had a chlorhexidine digluconate added for this purpose (Wiśniewska-Wrona–pers. comm).

Secondly, carbohydrate biopolymers are obtained from various sources which are naturally quite variable. A source organism, its geographic origin and the time of harvest affect the content and chemical structure of these polymers [19]. The extraction and purification techniques of these polysaccharides are not fully standardized and contribute to the variability of the end product [124]. As a result, the commercially available carbohydrate biopolymers have broadly specified physico-chemical characteristics and tests of their bioactivity do not always yield reproducible results ([19], Wiśniewska-Wrona—unpublished data). The high level of structural heterogeneity and polydispersity of these polymers make it difficult to provide a proven information on their efficiency, safety for the consumers and the environment, which is a huge disadvantage when going through the approval and commercialization process [125].

Thirdly, purification of carbohydrate polymers from natural raw materials is not very efficient and can be costly. For example, the traditional method of producing chitosan on an industrial scale from crustacean shell waste involves few chemical treatments in order to remove proteins, mineral salts and pigments. Then chitosan is obtained by hydrolysis of acetamide groups by severe alkaline treatment. The whole process is considered to be expensive and laborious; it also harmful to the environment as it requires using harsh chemicals and generates high amounts of wastes [126]. However, due to the growing demand on this polymer worldwide the global market of chitosan is developing dynamically, therefore alternative sources of this polymer (fungi, insects) and more sustainable production methods are being explored [127,128].

When comparing the three biopolymers included in the review among each other, we can point few clear differences in terms of their properties and applications. Chitosan is frequently treated as antifungal agent because of its proven direct antifungal activity, although it is also rather expensive compared to the other two polymers. Alginate is frequently combined with biocontrol bacteria and fungi, because due to its hydrophilic nature this polymer increases survival of these microorganisms. Cellulose is valued as the cheapest and commonly available resource. Although it cannot be used without modification because of its low solubility in water and lack of functional groups, therefore esters and ethers of this polymer are usually used.

## 6. Future Perspectives and Challenges

As we outlined in the introduction, the widespread use of chemical fungicides had a negative impact on environment, non-target organisms and human health. The growing concerns about these issues have led to the implementation of regulations restricting the use of these agrochemicals. For example, the current “Farm to Fork Strategy” aims at a significant reduction in pesticides use by 2030. Apart from regulatory framework, the consumers’ demand on healthier agricultural products provided a motivation to develop organic agriculture and stimulated development of plant protection products based on natural alternatives to chemical pesticides. As we showed in this review, carbohydrate biopolymers such as chitosan, alginate, cellulose or their derivatives, are suitable for this purpose because of their above-mentioned advantages including nontoxicity, biocompatibility and biodegradability. A lot of recently published studies showed that these compounds are versatile resources for producing plant protection formulations effective against pathogenic fungi because they can act as antifungal compounds, effective elicitors, carriers or matrices for controlled release of active ingredients. The growing interest in reducing the use of chemical pesticide may provide a motivation to improve and standardize production methods of carbohydrate biopolymers and to overcome commercialization barriers for the plant protection products containing these compounds.

Antifungal activity of the formulations based on carbohydrate biopolymers is usually lower or comparable to standard fungicidal treatments [46,51]. However, it can be enhanced by developing complex, more effective formulations which combine these biopolymers with other antifungal agents (Figure 2). Rapid development of nanotechnology opened possibilities of creating new promising forms of plant protection products based on nanoparticles, nano-/micro-carriers and electrospun nanofibers. However, there is a recognized need to evaluate these solutions for their safety and toxicity before they are introduced for use in agriculture [16].

The three carbohydrate biopolymers were successfully used to develop natural plant protection methods for various stages of plant production. As we showed in numerous examples, each of the three biopolymers can be used in treatments dedicated for seed sowing material, field applications and protection of harvested fruits and vegetables.

## Data Availability

Not applicable.
